# The Effect of Medical Student Volunteering in a Student-Run Clinic on Specialty Choice for Residency

**DOI:** 10.7759/cureus.967

**Published:** 2017-01-09

**Authors:** Ashley Brown, Rahim Ismail, Glenn Gookin, Caridad Hernandez, Grace Logan, Magdalena Pasarica

**Affiliations:** 1 College of Medicine, University of Central Florida; 2 Medical Education, University of Central Florida College of Medicine

**Keywords:** student-run free clinic, medical students, specialty choice, education, primary care, underserved, uninsured, volunteering, academic outcomes, academic success

## Abstract

Introduction: Student-run free clinics (SRFCs) are a recent popular addition to medical school education, and a subset of studies has looked at the influence of SRFC volunteering on the medical student’s career development. The majority of the research done in this area has focused on understanding if these SRFCs produce physicians who are more likely to practice medicine in underserved communities, caring for the uninsured. The remainder of the research has investigated if volunteering in an SRFC influences the specialty choice of medical school students. The results of these specialty choice studies give no definitive answer as to whether medical students chose primary or specialty care residencies as a result of their SRFC experience.

Keeping Neighbors in Good Health through Service (KNIGHTS) is the SRFC of the University of Central Florida College of Medicine (UCF COM). Both primary and specialty care is offered at the clinic. It is the goal of this study to determine if volunteering in the KNIGHTS SRFC influences UCF COM medical students to choose primary care, thereby helping to meet the rising need for primary care physicians in the United States.

Methods: A survey was distributed to first, second, and third-year medical students at the UCF COM to collect data on demographics, prior volunteering experience, and specialty choice for residency. Responses were then combined with records of volunteer hours from the KNIGHTS Clinic and analyzed for correlations. We analyzed the frequency and Pearson’s chi-squared values. A p value of less than 0.05 was considered statistically significant.

Results: Our survey had a total response rate of 39.8%. We found that neither the act of becoming a KNIGHTS Clinic volunteer nor the hours volunteered at the KNIGHTS Clinic influenced the UCF COM student’s choice to enter a primary care specialty (p = NS). Additionally, prior volunteering/clinical experience or the gender of the medical school student did not influence a student’s choice to volunteer at the KNIGHTS Clinic.

Discussion: Volunteering at KNIGHTS Clinic did not increase student choice to enter primary care, with students choosing other specialties at equal rates, probably due to the variety of specialties present at the KNIGHTS Clinic. This suggests that the volunteer attending physicians present at an SRFC may influence the choice of residency for students. It also suggests that SFRCs are not a viable tool to increase the number of primary care doctors in the United States.

## Introduction

Among medical schools across the United States (US), a recent trend has been to incorporate student-run free clinics (SRFC) into medical school education programs. A 2014 survey showed that there were 106 clinics operating within US Association of American Medical Colleges (AAMC) medical schools [1-2]. This represents a more than double increase in US AAMC medical school SRFCs since the last survey conducted in 2005 [2-3]. Studies have shown that the act of volunteering in an SRFC improves medical student attitudes towards the underserved, leadership, problem-solving, interprofessional attitudes, and even improves their academic scores [4-9]. Additionally, the mere presence of an SRFC in a medical school can strengthen the diversity of a medical school class [10]. Therefore, it has been suggested that SRFCs are a possible tool to educate student volunteers to be better equipped to face the problems of today’s healthcare system.

A recent report by the US Department of Health and Human Services predicts a shortage of 20,400 primary care physicians by 2020 due to both an increase in demand and a decrease in the number of graduating medical school students choosing to become primary care physicians [11]. SRFCs appear to shift a medical school’s mission toward a more community service focus and to increase longitudinal primary care experiences among the students, making them an ideal focus for research on student volunteer effects and also a possible recruitment tool for primary care.

Compared to studies on empathy toward underserved populations, specialty choice has been studied to a lesser extent, and the methods for analysis have varied widely, leading to contradictory results [7-8, 12-14]. The first study, in chronological order, showed that more SRFC volunteers matched in primary care residencies compared to non-volunteers. However, the volunteers at this clinic were a small minority (about 10%) of the medical school class and the overwhelming majority of volunteers (96.5%) chose a primary care specialty, making it likely that the volunteer population was not representative of the medical school as a whole [13]. Another study with a different methodology found no correlation between the presence of an SRFC at a medical school and the percentage of graduates who choose primary care. Even when corrected for characteristics of schools (urban, private, with/without a family medicine department) which have been shown to have an influence on students’ specialty choice, similar results were obtained [12]. The third study again examined the correlation between SRFC involvement and the individual match results into primary care specialties. This study found no statistically significant differences in the rates of students matching into a primary care specialty when comparing volunteers to non-volunteers. In this study, the authors did note that volunteers entered into primary care specialties at a greater rate, but that there usually was such a high percentage of the class entering primary care that the SRFC may have had an unmeasurable effect [8]. Another study analyzed results from paired pre-volunteering and post-volunteering surveys of first and second year SRFC volunteers on their future interest in being a primary care physician. It showed significant differences in the desire to practice as a primary care physician before and after volunteering at the SRFC [7]. The most recent study examined SRFC involvement correlation with the self-reported specialty choice of the volunteers over a span of 10 years. There was no significant difference in the tendency towards a primary care specialty choice in volunteers compared to non-volunteers. However, 26% of students who entered primary care reported to do so because of their experiences volunteering at the SRFC [14]. Additionally, of note, these studies usually failed to mention the percentage of primary care versus specialty care offered, which we suspect may have been one factor that influenced the range of conclusions.

Due to the contradictions and limitations of these studies, we conducted this study to examine SRFC volunteerism and its relationship with a student volunteer’s specialty choice, especially primary care, at the Keeping Neighbors in Good Health through Service (KNIGHTS) Clinic, the SRFC at the University of Central Florida College of Medicine (UCF COM). This clinic offers both primary and specialty care. Since the need for US primary care physicians is increasing, we aimed also to determine if our SRFC is a good way to recruit our students to primary care residency positions.

## Materials and methods

### SRFC description

Our study was conducted at the KNIGHTS Clinic, the SRFC of the UCF COM. At the time our survey was distributed, the KNIGHTS Clinic had 222 student volunteers (46 first year volunteers, 66 second year volunteers, 51 third year volunteers, 31 fourth year volunteers, and 28 University of Florida pharmacy students) and 21 physician volunteers (11 internal/family medicine, three hematology/oncology, three cardiology, one ophthalmology, one OB/GYN, one nutrition education, and one nephrology physician). The KNIGHTS Clinic operates for three hours every other week out of the facility of Grace Medical Home, a community free clinic in Orlando, Florida. The clinic has been able to offer 194 appointments to 47 current patients with complete follow-up care, including follow-up appointments and outside referrals. A standard clinic night comprises of two to three physician volunteers, 20 to 30 student volunteers, and six to 12 patients. Each clinic night a student is assigned to one of eight roles within the clinic, including Floor Manager, Front Desk, Care Coordination, Laboratory, Patient Education, Pharmacy, Electronic Medical Records, Community Referrals, and Patient Pairs as described in Table 1.

**Table 1:** Weighted Volunteer Hours Patient Pair: A combination of two medical students who together serve the role as the patient’s physician during the clinic night.

Clinic Position	Position Description	Patient Contact (hours/night)
Front Desk	Check in all patients for their appointments and alert the patient pairs when their patient arrives.	1
Patient Education	Offer optional one-on-one education to patients on a variety of topics, including but not limited to diet, exercise, smoking cessation, and medication.	3
Patient Pair	Conduct history, physical, and coordinate all care for the patient they are assigned to.	4
Floor Manager	Coordinate all operations of clinic night, including scheduling, clinic flow, and ensure that all patients receive quality care for each appointment at KNIGHTS Clinic.	4
Laboratory	Provide blood draws, pap smears, urinalysis, immunization, and EKG services to patients when needed.	1
Pharmacy	Provide medications from an in-clinic sample dispensary, education on medications and their side effects, and complete applications for patients to prescription assistance programs (PAP) to obtain low-cost or free prescription drugs from pharmaceutical companies.	2
Community Referrals	Orient new physician volunteers to the clinic and coordinate all outside referrals offered to our patients.	1
Electronic Medical Records	Assist the patient pair and volunteer physician in recording the patient visit note into our electronic medical record system.	3
Care Coordination	Coordinate continuity of care for each appointment visit, including reminder phone calls before the appointment, provide the patient with a treatment plan to take home, keep track of all labs and outside referrals that a patient is provided, and arrange contact between the patient pair and patient when results of labs and referrals come back.	3

### Survey distribution

This study analyzed 13 specific questions within a larger survey of 32 questions that looked at the demographics, satisfaction, prior clinical experience, KNIGHTS Clinic volunteer status, and specialty choice. The questions that were used in our analysis are included in the Appendix. Specialty choice was self-reported. The survey was released and written informed consent was obtained using the online survey software, Qualtrics (Qualtrics, LLC, Provo, UT 2014). The survey was created using sections of other surveys previously distributed to medical students for various reasons, including the 2014 Graduation Questionnaire distributed by the AAMC, and questions were modified to fit our study objectives [7]. The recruitment to this study was via email and the social media platform Facebook to all currently enrolled first (n = 123), second (n = 120), and third year (n = 96) medical students at the UCF COM in Orlando, FL. Data was collected from November 2014 to December 2014. The study was approved by the Institutional Review Board at the University of Central Florida (approval #SBE-14-10495). Participation in this study was strictly on a volunteer basis. Participants received $5 (US) compensation for the time spent completing the survey.

### Volunteer hour calculations

The total hours volunteered by each participant was recorded and combined with each volunteer’s survey results for data analysis. Two different variables were created from this data. Total volunteer hours were calculated as four hours for each clinic night volunteered. Weighted hours were calculated by quantifying the patient contact experienced during each night of volunteering at the clinic, according to the volunteer’s role served during that clinic night, and totaling all those individual hours over time. Description of the roles and the distribution of hours for the weighted hour's analysis are listed in Table 1.

### Statistical analysis

Survey results were analyzed for demographics, prior clinical experiences, volunteer status, and specialty choice. The statistical program SPSS 22.0 (IBM Corp., Armonk, NY) was used to analyze the frequency and Pearson’s chi-squared values. Frequency was measured to determine the distribution of the years in medical school, gender, race, age, undergraduate institution type, volunteer status, and prior experiences. The chi-squared analysis was used to determine the correlation between volunteer status and gender, prior experiences in medical school, and specialty choice.

## Results

All categorical data, including demographic information, is represented in number and percentage and all continuous data is represented with means and standard deviations. A P value of less than 0.05 was considered statistically significant. Our survey had a response rate of 39.8%. The results of our demographic analysis are displayed in Table 2. Data was collected from 47 first-year, 70 second-year, and 18 third-year UCF COM students. Second-year students comprised more than half of our sample (51.90%). The sample was evenly distributed between gender with 67 of those sampled being male and 68 being female. Whites comprised most of our sample (60.70%). The mean age was 24.56 years. Finally, most respondents attended a public undergraduate institution (74.10%).

**Table 2:** Description of Survey Volunteer Demographics Year in medical school, gender distribution, race, and type of undergraduate institution are presented as frequencies (percentages). Age is presented as a mean (standard deviation).

	Total Survey Participants (n = 135)
Year in Medical School	1^st^ year: 47 (34.80%); 2^nd^ year: 70 (51.90%); 3^rd^ year: 18 (13.30%)
Gender	Male: 67 (49.60%); Female: 68 (50.40%)
Race/Ethnicity	Asian/Pacific Islander: 40 (29.60%); Hispanic: 8 (5.90%); White: 82 (60.70%); Other: 5 (3.70%)
Age	Mean: 24.56 years (2.56 yrs)
Undergraduate Institution	Public: 100 (74.10%); Private: 35 (25.90%)

The sample was then analyzed in regards to clinical experiences before medical school to see if there was any effect on the decision to become an SRFC volunteer with the results contained in Table 3. Of the 135 students sampled, 79 were KNIGHTS Clinic volunteers while 56 were not. KNIGHTS Clinic volunteers had higher rates of prior experience delivering clinical care, prior administration of study interventions for clinical research, and prior experience conducting clinical interviews or tests for clinical research. Non-KNIGHTS Clinic volunteers had higher rates of the other three variables. However, we found that none of these differences were significant on chi-squared analysis (p = NS), making prior experiences evenly distributed among KNIGHTS Clinic volunteers compared to non-volunteers. We conclude that prior experiences have no effect on whether a person volunteers at the UCF COM KNIGHTS Clinic. This suggests that upon entering medical school, the populations of KNIGHTS Clinic volunteers compared to non-KNIGHTS Clinic volunteers are essentially the same in regards to prior clinical experiences.

**Table 3:** Description of Previous Volunteering and Clinical Experience Data presented in frequencies (percentages). P-value for the Chi-squared test is presented in the far column.

	KNIGHTS Clinic volunteer (n=79)	NOT a KNIGHTS Clinic volunteer (n=56)	Chi-Squared Analysis
PRIOR experience delivering clinical care (n = 135)	38 (48.10%)	25 (44.60%)	p = 0.69
PRIOR employment or held a degree as a health care professional (n = 134)	13 (16.50%)	12 (21.40%)	p = 0.43
PRIOR providing of direct clinical care for 3+ months (n = 134)	16 (20.30%)	17 (30.40%)	p = 0.19
PRIOR administration of study interventions for clinical research (n = 134)	12 (15.20%)	6 (10.70%)	p = 0.43
PRIOR conducting of clinical interviews/tests for clinical research (n = 131)	11 (13.90%)	7 (12.50%)	p = 0.77
PRIOR participation in volunteer clinical experiences (n = 135)	67 (84.80%)	48 (85.70%)	p = 0.88

When KNIGHTS Clinic volunteers and non-volunteers were analyzed for their choice to specialize in primary care, defined as Family Medicine, General Internal Medicine, Internal Medicine/Pediatrics, or Pediatrics, higher rates of volunteers planned to enter primary care but the difference was not statistically significant as shown in Table 4. This result held true when we looked at volunteers with clinical experience prior to medical school compared to those with no prior volunteer clinical experience (p = NS). This allowed us to conclude that the act of volunteering for the KNIGHTS Clinic had no association with their intention to apply to a primary care specialty over a different specialty and that prior clinical experience was not a significant influencing variable.

**Table 4:** Volunteering Effect on Specialty Choice Data presented in frequencies (percentages). P-value for the Chi-squared test is presented in the far column.

	KNIGHTS Clinic volunteer (n=78)	NOT a KNIGHTS Clinic volunteer (n=56)	Chi-squared Analysis
Planning on Choosing a Primary Care Residency	46 (58.97%)	25 (44.64%)	p = 0.10

We then analyzed whether KNIGHTS Clinic volunteers with higher volunteer hours entered primary care specialties at a higher rate than volunteers with a lower number of hours. We looked at the total volunteer hours and at the weighted hours. We calculated the mean total hours to be 16.20 (standard deviation (SD): 16.01) and mean weighted hours to be 10.75 (SD: 10.75). Using that information, volunteers were divided into four groups: volunteers that had total volunteer hours higher than the mean (equal or more than 17 hours), volunteers that had total volunteer hours lower than the mean (0 - 16 hours), volunteers that had weighted volunteer hours higher than the mean (equal or more than 11 hours), and volunteers that had weighted volunteers hours lower than the mean (0 - 10 hours).

The KNIGHTS Clinic volunteers with higher total hours than the mean were not significantly more likely to plan to enter a primary care specialty than those with lower hours (p = NS) as shown in Table 5. Similarly, those KNIGHTS Clinic volunteers with higher weighted hours than the mean were not more likely to plan to enter a primary care specialty than those with lower hours (p = NS), also shown in Table 5.

**Table 5:** KNIGHTS Clinic Volunteer Specialty Choice by Hours Data presented as frequencies (percentages)

	Primary Care Specialty Choice
Low Total Hours (n = 50)	29 (58.00%)
High Total Hours (n = 28)	17 (60.71%)
Low-Weighted Hours (n = 43)	25 (58.14%)
High-Weighted Hours (n = 35)	21 (60.00%)

We also analyzed this excluding volunteers with zero total volunteer hours. There was a slight change in the mean total hours to 19.69 (SD: 15.57) and the mean weighted hours to 12.86 (SD: 10.61) when excluding these volunteers; however, neither had statistically significant differences for either total (p = NS) or weighted hours (p = NS) in regards to the KNIGHTS Clinic volunteer's plan to enter a primary care specialty compared to a different specialty. We conclude that the number of volunteer hours at KNIGHTS Clinic or the amount of patient contact experienced during the KNIGHTS Clinic did not influence the likelihood of a KNIGHTS Clinic volunteer to choose a primary care specialty compared to a different specialty.

As the last step, we conducted an analysis of the gender distribution of our KNIGHTS Clinic volunteers compared to the hours they spent at the clinic. We found that neither total hours (p = NS) nor weighted hours (p = NS) were significant for gender differences as shown in Table 6. This allowed us to conclude that the gender of the volunteer does not determine the amount of time they volunteer at KNIGHTS Clinic or their specialty choice.

**Table 6:** KNIGHTS Clinic Volunteer Specialty Choice by Hours and Sex Data presented as frequencies (percentages)

	Male	Female
Low Total Hours (n = 51)	26 (51.98%)	25 (49.01%)
High Total Hours (n = 28)	12 (42.86%)	16 (57.14%)
Low Weighted Hours (n = 44)	21 (47.72%)	23 (52.27%)
High Weighted Hours (n = 35)	17 (48.57%)	18 (51.42%)

## Discussion

We found no significant association between a medical school student’s choice to enter a primary care specialty (applying for primary care residency) and volunteering at the UCF COM KNIGHTS Clinic. Even when looking at specific characteristics of the KNIGHTS clinic volunteers like gender, prior clinical/volunteering experiences, total time spent at KNIGHTS Clinic, and patient contact hours at KNIGHTS Clinic, there were no significant associations between those factors and a student’s choice to pursue a primary care specialty. Our study agrees with the results generated by Tong, et al. in 2012, Vaikunth, et al. in 2014, and Weinreich, et al. in 2015 but disagrees with the other two studies done on the topic. Furthermore, while three of the past studies found that female gender either makes a person more likely to become a volunteer at an SRFC or more likely to appreciate SRFC volunteering, our study did not find a correlation between volunteering and female gender [6, 8, 14]. This also disagrees with past research that states that female gender influences specialty choice [15]. While this was not a purpose of our study, it is an interesting finding and is worthy of further investigation.

While our results are conclusive, our study has several limitations. First, our study was heavily weighted with second-year medical students, likely due to the point in the academic year that our survey was released. Therefore, our results may not be representative of the entire population of KNIGHTS Clinic volunteers. In the future, the study will be surveying clinic volunteers at a different point in the academic year to make the sample more representative.

Secondly, our specialty choice data originates from a self-reported survey rather than match results from practicing alumni statistics [8, 12-13]. On one hand, this may more accurately display “specialty choice” rather than the specialty that one ends up in due to cost, location, competition, or other complicating factors. However, because our survey was mostly second-year students, there is a possibility that the student’s specialty choice changed by the time they reached the fourth year.

Finally, it is worth mentioning that even though our SRFC provides more primary care visits than specialty care, it does expose students to a fair amount of specialists. Working with the specialists in the SRFC may have increased the students’ interests in those specialties just as exposure to a primary care physician may increase interest in primary care. In addition, at our school, primary care faculty are underrepresented compared with specialists. A future study could look at the student choice for the specialties offered in the clinic, in addition to primary care.

## Conclusions

Our study illustrates that students from many different backgrounds and with very different futures volunteer their time with the KNIGHTS Clinic. Volunteering at KNIGHTS Clinic did not increase students’ choice to enter into primary care, with students choosing other specialties at equal rates, possibly influenced by the presence of non-primary care specialties at the KNIGHTS Clinic. This suggests that the volunteer attending physicians offered within the SRFC may impact the choice of residency for students. It also suggests that SRFCs are not a viable tool to increase the number primary care doctors in the US.

## Appendices

Survey Questions

1. Year in Medical School

- M1
- M2
- M3
- M4

2. Gender

- Male
- Female
- Other

3. Race and Ethnicity

- African American
- Asian/Pacific Islander
- Hispanic
- White
- Other

4. Please indicate your age (free text box)

5. What type of undergraduate institution did you attend?

- Private
- Public

6. PRIOR Medical School: Did you have experience delivering clinical care (non-shadowing such as interviewing patients, administering clinical tests, etc)?

- Yes
- No

7. PRIOR Medical School: Where you employed or held a degree as a health profession (EMT, BSN, PA, CNA, RN, etc.)?

- Yes
- No

8. PRIOR Medical School: Did you provide direct clinical care for 3+ months at any capacity?

- Yes
- No

9. PRIOR Medical School: Did you administer study interventions for clinical research?

- Yes
- No

10. PRIOR Medical School: Did you conduct clinical interviews/tests for clinical research?

- Yes
- No

11. PRIOR Medical School: Did you participate in volunteer clinical experiences?

- Yes
- No

12. Are you a KNIGHTS Clinic volunteer?

- Yes
- No

13. Are you planning on specializing in Family Medicine, General Internal Medicine, Internal Medicine/Pediatrics or Pediatrics?

- Yes
- No
